# *catena*-Poly[[diaqua­nickel(II)]-bis­(μ-2-{[5-(pyridin-4-yl)-1,3,4-oxadiazol-2-yl]sulfan­yl}acetato)]

**DOI:** 10.1107/S1600536812020259

**Published:** 2012-05-12

**Authors:** Ru-Qin Gao, Chao-Hui Xia, Guo-Ting Li

**Affiliations:** a Department of Environmental and Municipal Engineering, North China University of Water Conservancy and Electric Power, Zhengzhou 450011, People’s Republic of China; b Henan Vocational College of Chemical Technology, Zhengzhou 450052, People’s Republic of China

## Abstract

In the title compound, [Ni(C_9_H_6_N_3_O_3_S)_2_(H_2_O)_2_]_*n*_, the Ni^II^ atom, located on an inversion center, is ligated in an octa­hedral geometry by two carboxyl­ate O atoms from two 2-{[5-(pyridin-4-yl)-1,3,4-oxadiazol-2-yl]sulfan­yl}acetate (*L*) ligands and two O atoms from water mol­ecules in the equatorial plane, and two pyridine N atoms from other two *L* ligands at the apical sites. Two *L* ligands bridge pairs of metal atoms in an anti­parallel manner, forming centrosymmetric dinuclear quasi-recta­ngular units which are linked into infinite double-stranded chains parallel to [100]. O—H⋯O hydrogen bonds between the coordinating water mol­ecules and the carboxyl­ate groups of the *L* ligand as well as interchain S⋯N inter­actions [2.726 (2)–3.363 (2) Å] lead to the formation of a layer structure parallel to (001).

## Related literature

For coordination polymers of 1,3,4-oxadiazole-2-thione, see: Wu *et al.* (2010 ▶); Lundin *et al.* (2006 ▶); Wang *et al.* (2007) ▶. For coordination polymers of symmetric pyridyl-containing oxadiazole ligands, see: Ma *et al.* (2007 ▶); Du *et al.* (2006) ▶. For unsymmetric pyridyl-containing oxadiazole ligands, see: Wang & Li (2011 ▶). 
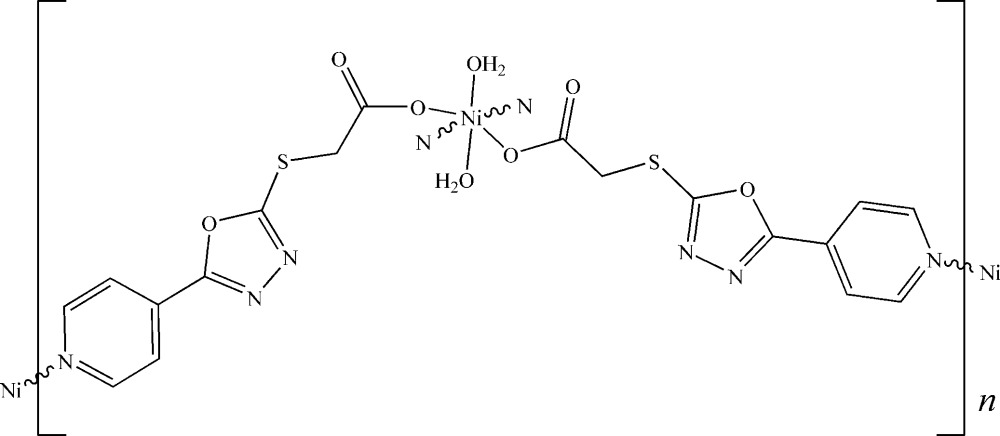


## Experimental

### 

#### Crystal data

[Ni(C_9_H_6_N_3_O_3_S)_2_(H_2_O)_2_]
*M* *_r_* = 567.20
Monoclinic, 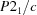

*a* = 11.8862 (18) Å
*b* = 5.6431 (9) Å
*c* = 15.500 (2) Å
β = 95.687 (2)°
*V* = 1034.5 (3) Å^3^
*Z* = 2
Mo *K*α radiation
μ = 1.20 mm^−1^
*T* = 293 K
0.15 × 0.13 × 0.07 mm

#### Data collection

Siemens SMART CCD diffractometer
7195 measured reflections
1822 independent reflections
1488 reflections with *I* > 2σ(*I*)
*R*_int_ = 0.034

#### Refinement

*R*[*F* ^2^ > 2σ(*F* ^2^)] = 0.028
*wR*(*F* ^2^) = 0.068
*S* = 1.03
1822 reflections
166 parameters
2 restraints
H atoms treated by a mixture of independent and constrained refinement
Δρ_max_ = 0.25 e Å^−3^
Δρ_min_ = −0.28 e Å^−3^

### 

Data collection: *SMART* (Siemens, 1996 ▶); cell refinement: *SAINT* (Siemens, 1994 ▶); data reduction: *SAINT*; program(s) used to solve structure: *SHELXS97* (Sheldrick, 2008 ▶); program(s) used to refine structure: *SHELXL97* (Sheldrick, 2008 ▶); molecular graphics: *SHELXTL* (Sheldrick, 2008 ▶); software used to prepare material for publication: *SHELXL97*.

## Supplementary Material

Crystal structure: contains datablock(s) I, global. DOI: 10.1107/S1600536812020259/zj2075sup1.cif

Structure factors: contains datablock(s) I. DOI: 10.1107/S1600536812020259/zj2075Isup2.hkl

Additional supplementary materials:  crystallographic information; 3D view; checkCIF report

## Figures and Tables

**Table 1:** Selected bond lengths (Å)

Ni1—O2	2.0702 (16)
Ni1—O4	2.0781 (18)
Ni1—N1^i^	2.1157 (19)

Symmetry code: (i) 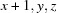.

**Table 2:** Hydrogen-bond geometry (Å, °)

*D*—H⋯*A*	*D*—H	H⋯*A*	*D*⋯*A*	*D*—H⋯*A*
O4—H4*A*⋯O3	0.82 (1)	1.83 (1)	2.633 (3)	167 (3)
O4—H4*B*⋯O2^ii^	0.82 (1)	2.11 (2)	2.857 (3)	153 (3)

Symmetry code: (ii) 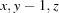.

**Fig. 1.**
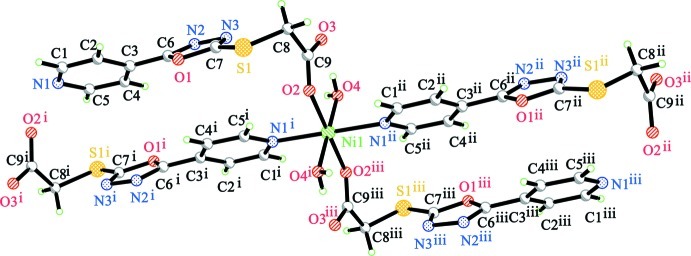
Coordination environment of the nickel atom in (1). Displacement ellipsoids are drawn at the 30% probability level. Symmetry code: (i) -x + 1, -y, -z + 1; (ii) x + 1, y, z; (iii) -x, -y, -z + 1.

**Fig. 2.**
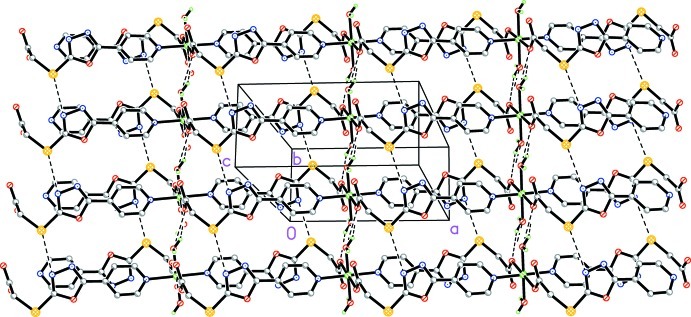
View of the two-dimensional network structure in (1) formed by interchain S···N interactions and multiple O—H···O hydrogen-bonding interactions

## supplementary crystallographic information

### Comment

There have been considerable interests in the coordination polymers of 1,3,4-oxadiazole-2-thione because of their intriguing architectures (Wu, *et al.*, 2010) and potential applications as functional materials (Lundin, *et al.*, 2006; Wang, *et al.*, 2007). In particular, pyridyl-containing oxadiazole ligands, such as symmetric 5-phenyl-1,3,4-oxadiazole-2-thione (Ma, *et al.*, 2007) and 5-(4-pyridyl)-1,3,4-oxadiazole-2-thione (Du, *et al.*, 2006), have been extensively explored in the construction of porous coordination polymers. As our continuous work in this aspect (Wang & Li, 2011), we report that the reaction of NiCl_2_.6H_2_O and sodium(I) salt of 2-(5-(pyridin-4-yl)-1,3,4-oxadiazol-2-ylthio)acetic acid (HL) leads to a new complex [Ni(*L*)_2_(H_2_O)_2_]_n_ (1) herein.

In (1) the Ni^II^ center is located at the inversion center ligated by two carboxylato O atoms from two deprotonated *L* and two O atoms from water molecules in the equatorial plane, and two pyridyl N atoms from other two deprotonated *L* at the apical sites. Thus the Ni^II^ ion is in a six-coordinated octahedral coordination geometry (Fig. 1). The bond distances of Ni—O and Ni—N range from 2.070 (2) to 2.116 (2) Å, while O—Ni—N angles range from 85.90 (7) to 94.10 (7) °, indicating a slight distortion from an ideal octahedron.

Complex (1) displays an extended infinite double-strand chain structure constructed of dinuclear quasi-rectangle units (Fig. 2). The dinuclear quasi-rectangle units are centrosymmetric and formed by two *L* anions antiparallelly bridging two metal centers in monodentate modes with two nickel atoms and two methylene carbon atoms of the *L* at the corners and the diagonal Ni···Ni distances of 11.886 (2) Å. As for *L*, the pyridyl group and the acetate group deviate from the center ring of oxadiazole-2-thione group, with the dihedral angels being 36.0 (7) and 88.5 (7) °, respectively. Notably, the conformation of *L* is apt to the dinuclear quasi-rectangle which is further stabilized by CH···π stacking interactions between antiparallel the pyridyl-1,3,4-oxadiazol groups of the *L* in the same rectangle unit with the distances of H_pyridyl_ to centroid of oxadiazol group being 3.320 (2) Å and 3.353 (2) Å. The chains of complex (1) are connected by O—H···O hydrogen bonds between the coordinated water molecules (as donors) and the carboxylate groups of *L* (as acceptors), leading to the formation of a two-dimensional network structure (Fig. 2, Table 3). Additionally, the interchain weak interactions between S and N of the oxadiazole-2-thione groups of *L* stabilize the layer structure (the distances of S···N being in a range of 2.726 (2) to 3.363 (2) Å).

### Experimental

For the synthesis of sodium(I) salt of ligand 2-(5-(pyridin-4-yl)-1,3,4-oxadiazol-2-ylthio)acetic acid (HL), see: Wang & Li, (2011). The title compound (1).n(H_2_O) was prepared according to the following process. A mixture of NaL (51.8 mg, 0.2 mmol), NiCl_2_.6H_2_O (23.8 mg, 0.1 mmol) and deionized water (20 ml) was stirred for 30 minutes and then filtered. The filtrate was allowed to evaporate at room temperature for three days, and then green needle crystals were obtain in 72% yield. Selected IR (cm^-1^, KBr pellet): 3374(*m*), 3091(*w*), 2994(*w*), 1621(*m*), 1579(*s*), 1463(*s*), 1382(*s*), 1224(*m*), 1192(*m*), 1065(*m*), 958(*w*), 707(*s*), 586(*w*).

### Refinement

The H atoms of water were located from difference Fourier maps and included in the final refinement by using geometrical restrains, while the other hydrogen atom positions were generated geometrically and these H atoms were allowed to ride on their parent atoms.

### Crystal data

**Table:**

[Ni(C_9_H_6_N_3_O_3_S)_2_(H_2_O)_2_]	*F*(000) = 580
*M**_r_* = 567.20	*D*_x_ = 1.821 Mg m^−^^3^
Monoclinic, *P*2_1_/*c*	Mo *K*α radiation, λ = 0.71073 Å
Hall symbol: -p 2ybc	Cell parameters from 1733 reflections
*a* = 11.8862 (18) Å	θ = 2.6–24.8°
*b* = 5.6431 (9) Å	µ = 1.20 mm^−^^1^
*c* = 15.500 (2) Å	*T* = 293 K
β = 95.687 (2)°	Needle, pale green
*V* = 1034.5 (3) Å^3^	0.15 × 0.13 × 0.07 mm
*Z* = 2	

### Data collection

**Table:**

Siemens SMART CCD diffractometer	1488 reflections with *I* > 2σ(*I*)
Radiation source: fine-focus sealed tube	*R*_int_ = 0.034
Graphite monochromator	θ_max_ = 25.0°, θ_min_ = 2.6°
ω scan	*h* = −14→13
7195 measured reflections	*k* = −6→6
1822 independent reflections	*l* = −18→18

### Refinement

**Table:**

Refinement on *F*^2^	Primary atom site location: structure-invariant direct methods
Least-squares matrix: full	Secondary atom site location: difference Fourier map
*R*[*F*^2^ > 2σ(*F*^2^)] = 0.028	Hydrogen site location: inferred from neighbouring sites
*wR*(*F*^2^) = 0.068	H atoms treated by a mixture of independent and constrained refinement
*S* = 1.03	*w* = 1/[σ^2^(*F*_o_^2^) + (0.0266*P*)^2^ + 0.8247*P*] where *P* = (*F*_o_^2^ + 2*F*_c_^2^)/3
1822 reflections	(Δ/σ)_max_ < 0.001
166 parameters	Δρ_max_ = 0.25 e Å^−^^3^
2 restraints	Δρ_min_ = −0.28 e Å^−^^3^

### Special details

**Table:**

Geometry. All e.s.d.'s (except the e.s.d. in the dihedral angle between two l.s. planes) are estimated using the full covariance matrix. The cell e.s.d.'s are taken into account individually in the estimation of e.s.d.'s in distances, angles and torsion angles; correlations between e.s.d.'s in cell parameters are only used when they are defined by crystal symmetry. An approximate (isotropic) treatment of cell e.s.d.'s is used for estimating e.s.d.'s involving l.s. planes.
Refinement. Refinement of *F*^2^ against ALL reflections. The weighted *R*-factor *wR* and goodness of fit *S* are based on *F*^2^, conventional *R*-factors *R* are based on *F*, with *F* set to zero for negative *F*^2^. The threshold expression of *F*^2^ > σ(*F*^2^) is used only for calculating *R*-factors(gt) *etc*. and is not relevant to the choice of reflections for refinement. *R*-factors based on *F*^2^ are statistically about twice as large as those based on *F*, and *R*- factors based on ALL data will be even larger.

### Fractional atomic coordinates and isotropic or equivalent isotropic displacement parameters (Å^2^)

**Table:**

	*x*	*y*	*z*	*U*_iso_*/*U*_eq_	
Ni1	0.5000	0.0000	0.5000	0.01764 (14)	
S1	0.22867 (5)	0.52524 (11)	0.32025 (4)	0.02534 (17)	
O1	0.03642 (14)	0.3493 (3)	0.36616 (11)	0.0251 (4)	
N1	−0.33708 (16)	0.0597 (4)	0.46039 (13)	0.0214 (5)	
C1	−0.3094 (2)	0.2565 (5)	0.41861 (17)	0.0267 (6)	
H1	−0.3640	0.3791	0.4099	0.032*	
O2	0.42671 (14)	0.2203 (3)	0.40344 (10)	0.0223 (4)	
N2	0.04253 (19)	−0.0307 (4)	0.33372 (17)	0.0349 (6)	
C2	−0.2055 (2)	0.2898 (5)	0.38770 (18)	0.0285 (6)	
H2	−0.1895	0.4322	0.3586	0.034*	
O3	0.41911 (15)	−0.0211 (3)	0.28832 (12)	0.0307 (4)	
N3	0.14163 (18)	0.0782 (4)	0.30919 (16)	0.0329 (6)	
C3	−0.1253 (2)	0.1121 (5)	0.39981 (16)	0.0227 (6)	
O4	0.49432 (15)	−0.2945 (3)	0.41919 (12)	0.0258 (4)	
C4	−0.1526 (2)	−0.0926 (5)	0.44222 (16)	0.0254 (6)	
H4	−0.0996	−0.2183	0.4512	0.030*	
C5	−0.2588 (2)	−0.1107 (5)	0.47130 (16)	0.0229 (6)	
H5	−0.2769	−0.2515	0.5005	0.027*	
C6	−0.0151 (2)	0.1322 (5)	0.36561 (17)	0.0241 (6)	
C7	0.13385 (19)	0.2981 (5)	0.32989 (16)	0.0233 (6)	
C8	0.3334 (2)	0.3561 (5)	0.26933 (16)	0.0241 (6)	
H8A	0.2953	0.2784	0.2171	0.029*	
H8B	0.3895	0.4684	0.2495	0.029*	
C9	0.39724 (19)	0.1663 (5)	0.32486 (16)	0.0210 (5)	
H4B	0.459 (2)	−0.417 (3)	0.4250 (18)	0.032*	
H4A	0.470 (2)	−0.229 (5)	0.3738 (11)	0.032*	

### Atomic displacement parameters (Å^2^)

**Table:**

	*U*^11^	*U*^22^	*U*^33^	*U*^12^	*U*^13^	*U*^23^
Ni1	0.0149 (2)	0.0173 (2)	0.0210 (2)	0.00045 (18)	0.00284 (17)	0.00042 (19)
S1	0.0185 (3)	0.0219 (3)	0.0362 (4)	0.0001 (3)	0.0056 (3)	0.0036 (3)
O1	0.0187 (9)	0.0241 (10)	0.0338 (10)	0.0013 (8)	0.0087 (8)	−0.0002 (8)
N1	0.0174 (11)	0.0225 (11)	0.0245 (11)	−0.0009 (9)	0.0030 (9)	−0.0010 (9)
C1	0.0225 (14)	0.0194 (13)	0.0393 (16)	0.0029 (11)	0.0090 (12)	0.0014 (12)
O2	0.0231 (9)	0.0221 (9)	0.0215 (10)	0.0018 (7)	0.0011 (7)	0.0020 (7)
N2	0.0254 (12)	0.0278 (13)	0.0546 (16)	−0.0049 (11)	0.0187 (11)	−0.0056 (12)
C2	0.0277 (14)	0.0201 (14)	0.0388 (16)	−0.0006 (11)	0.0095 (12)	0.0058 (12)
O3	0.0350 (11)	0.0293 (11)	0.0281 (10)	0.0086 (9)	0.0040 (8)	−0.0047 (9)
N3	0.0207 (12)	0.0244 (13)	0.0563 (16)	−0.0032 (10)	0.0178 (11)	−0.0030 (11)
C3	0.0178 (12)	0.0250 (14)	0.0253 (14)	−0.0019 (11)	0.0016 (10)	−0.0022 (11)
O4	0.0288 (11)	0.0211 (10)	0.0271 (10)	−0.0007 (8)	0.0013 (8)	0.0001 (8)
C4	0.0212 (13)	0.0262 (14)	0.0284 (14)	0.0043 (11)	0.0006 (11)	0.0016 (12)
C5	0.0213 (13)	0.0242 (14)	0.0236 (14)	0.0002 (11)	0.0038 (11)	0.0027 (11)
C6	0.0178 (13)	0.0255 (14)	0.0293 (14)	−0.0020 (11)	0.0038 (11)	0.0020 (12)
C7	0.0144 (12)	0.0266 (15)	0.0291 (14)	0.0027 (11)	0.0036 (11)	0.0026 (12)
C8	0.0164 (13)	0.0307 (15)	0.0258 (14)	0.0006 (11)	0.0049 (11)	0.0050 (12)
C9	0.0121 (12)	0.0275 (14)	0.0244 (14)	−0.0027 (11)	0.0063 (10)	0.0033 (12)

### Geometric parameters (Å, º)

**Table:**

Ni1—O2^i^	2.0702 (16)	N2—C6	1.275 (3)
Ni1—O2	2.0702 (16)	N2—N3	1.414 (3)
Ni1—O4	2.0781 (18)	C2—C3	1.384 (3)
Ni1—O4^i^	2.0781 (18)	C2—H2	0.9500
Ni1—N1^ii^	2.1157 (19)	O3—C9	1.239 (3)
Ni1—N1^iii^	2.1157 (19)	N3—C7	1.287 (3)
S1—C7	1.723 (3)	C3—C4	1.383 (4)
S1—C8	1.811 (2)	C3—C6	1.465 (3)
O1—C7	1.367 (3)	O4—H4B	0.816 (10)
O1—C6	1.369 (3)	O4—H4A	0.819 (10)
N1—C5	1.337 (3)	C4—C5	1.385 (3)
N1—C1	1.343 (3)	C4—H4	0.9500
N1—Ni1^iv^	2.1157 (19)	C5—H5	0.9500
C1—C2	1.380 (4)	C8—C9	1.528 (3)
C1—H1	0.9500	C8—H8A	0.9900
O2—C9	1.271 (3)	C8—H8B	0.9900
			
O2^i^—Ni1—O2	180.00 (6)	C7—N3—N2	105.6 (2)
O2^i^—Ni1—O4	86.66 (7)	C4—C3—C2	118.6 (2)
O2—Ni1—O4	93.34 (7)	C4—C3—C6	119.8 (2)
O2^i^—Ni1—O4^i^	93.34 (7)	C2—C3—C6	121.6 (2)
O2—Ni1—O4^i^	86.66 (7)	Ni1—O4—H4B	127 (2)
O4—Ni1—O4^i^	180.0	Ni1—O4—H4A	98 (2)
O2^i^—Ni1—N1^ii^	88.50 (7)	H4B—O4—H4A	110 (3)
O2—Ni1—N1^ii^	91.50 (7)	C3—C4—C5	118.7 (2)
O4—Ni1—N1^ii^	85.90 (7)	C3—C4—H4	120.6
O4^i^—Ni1—N1^ii^	94.10 (7)	C5—C4—H4	120.6
O2^i^—Ni1—N1^iii^	91.50 (7)	N1—C5—C4	123.4 (2)
O2—Ni1—N1^iii^	88.50 (7)	N1—C5—H5	118.3
O4—Ni1—N1^iii^	94.10 (7)	C4—C5—H5	118.3
O4^i^—Ni1—N1^iii^	85.90 (7)	N2—C6—O1	113.0 (2)
N1^ii^—Ni1—N1^iii^	180.0	N2—C6—C3	128.2 (2)
C7—S1—C8	97.45 (12)	O1—C6—C3	118.9 (2)
C7—O1—C6	101.82 (19)	N3—C7—O1	113.0 (2)
C5—N1—C1	117.0 (2)	N3—C7—S1	129.2 (2)
C5—N1—Ni1^iv^	119.62 (16)	O1—C7—S1	117.79 (18)
C1—N1—Ni1^iv^	123.17 (17)	C9—C8—S1	116.64 (17)
N1—C1—C2	123.4 (2)	C9—C8—H8A	108.1
N1—C1—H1	118.3	S1—C8—H8A	108.1
C2—C1—H1	118.3	C9—C8—H8B	108.1
C9—O2—Ni1	127.26 (16)	S1—C8—H8B	108.1
C6—N2—N3	106.5 (2)	H8A—C8—H8B	107.3
C1—C2—C3	118.8 (2)	O3—C9—O2	126.3 (2)
C1—C2—H2	120.6	O3—C9—C8	117.1 (2)
C3—C2—H2	120.6	O2—C9—C8	116.5 (2)

Symmetry codes: (i) −*x*+1, −*y*, −*z*+1; (ii) *x*+1, *y*, *z*; (iii) −*x*, −*y*, −*z*+1; (iv) *x*−1, *y*, *z*.

### Hydrogen-bond geometry (Å, º)

**Table:**

*D*—H···*A*	*D*—H	H···*A*	*D*···*A*	*D*—H···*A*
O4—H4*A*···O3	0.82 (1)	1.83 (1)	2.633 (3)	167 (3)
O4—H4*B*···O2^v^	0.82 (1)	2.11 (2)	2.857 (3)	153 (3)

Symmetry code: (v) *x*, *y*−1, *z*.

## References

[bb1] Du, M., Zhang, Z. H., Zhao, X. J. & Xu, Q. (2006). *Inorg. Chem.* **45**, 5785–5792. 10.1021/ic060129v 16841982

[bb2] Lundin, N. J., Blackman, A. G., Gordon, K. C. & Officer, D. L. (2006). *Angew. Chem. Int. Ed.* **45**, 2582–2584. 10.1002/anie.200504532 16555347

[bb3] Ma, C., Tian, G. & Zhang, R. (2007). *Inorg. Chim. Acta*, **360**, 1762–1766.

[bb5] Sheldrick, G. M. (2008). *Acta Cryst.* A**64**, 112–122. 10.1107/S0108767307043930 18156677

[bb6] Siemens (1994). *SAINT* Siemens Analytical X-ray Instruments Inc., Madison, Wisconsin, USA.

[bb7] Siemens (1996). *SMART* Siemens Analytical X-ray Instruments Inc., Madison, Wisconsin, USA.

[bb8] Wang, H.-R. & Li, G.-T. (2011). *Acta Cryst.* E**67**, m1457. 10.1107/S1600536811038918 PMC3201331 22058719

[bb9] Wang, Y. T., Tang, G. M. & Quang, Z. W. (2007). *Polyhedron*, **26**, 4542–4550.

[bb10] Wu, B. L., Wang, R. Y., Ye, E., Zhang, H. Y. & Hou, H. W. (2010). *Inorg. Chem. Commun.* **13**, 157–159.

